# A Review on Human Activity Recognition Using Vision-Based Method

**DOI:** 10.1155/2017/3090343

**Published:** 2017-07-20

**Authors:** Shugang Zhang, Zhiqiang Wei, Jie Nie, Lei Huang, Shuang Wang, Zhen Li

**Affiliations:** ^1^College of Information Science and Engineering, Ocean University of China, Qingdao, China; ^2^Department of Computer Science and Technology, Tsinghua University, Beijing, China

## Abstract

Human activity recognition (HAR) aims to recognize activities from a series of observations on the actions of subjects and the environmental conditions. The vision-based HAR research is the basis of many applications including video surveillance, health care, and human-computer interaction (HCI). This review highlights the advances of state-of-the-art activity recognition approaches, especially for the activity representation and classification methods. For the representation methods, we sort out a chronological research trajectory from global representations to local representations, and recent depth-based representations. For the classification methods, we conform to the categorization of template-based methods, discriminative models, and generative models and review several prevalent methods. Next, representative and available datasets are introduced. Aiming to provide an overview of those methods and a convenient way of comparing them, we classify existing literatures with a detailed taxonomy including representation and classification methods, as well as the datasets they used. Finally, we investigate the directions for future research.

## 1. Introduction

Human activity recognition (HAR) is a widely studied computer vision problem. Applications of HAR include video surveillance, health care, and human-computer interaction. As the imaging technique advances and the camera device upgrades, novel approaches for HAR constantly emerge. This review aims to provide a comprehensive introduction to the video-based human activity recognition, giving an overview of various approaches as well as their evolutions by covering both the representative classical literatures and the state-of-the-art approaches.

Human activities have an inherent hierarchical structure that indicates the different levels of it, which can be considered as a three-level categorization. First, for the bottom level, there is an atomic element and these action primitives constitute more complex human activities. After the action primitive level, the action/activity comes as the second level. Finally, the complex interactions form the top level, which refers to the human activities that involve more than two persons and objects. In this paper, we follow this three-level categorization namely action primitives, actions/activities, and interactions. This three-level categorization varies a little from previous surveys [1–4] and maintains a consistent theme. Action primitives are those atomic actions at the limb level, such as “stretching the left arm,” and “raising the right leg.” Atomic actions are performed by a specific part of the human body, such as the hands, arms, or upper body part [4]. Actions and activities are used interchangeably in this review, referring to the whole-body movements composed of several action primitives in temporal sequential order and performed by a single person with no more person or additional objects. Specifically, we refer the terminology human activities as all movements of the three layers and the activities/actions as the middle level of human activities. Human activities like walking, running, and waving hands are categorized in the actions/activities level. Finally, similar to Aggarwal et al.'s review [2], interactions are human activities that involve two or more persons and objects. The additional person or object is an important characteristic of interaction. Typical examples of interactions are cooking which involves one person and various pots and pans and kissing that is performed by two persons.

**Figure 1:**
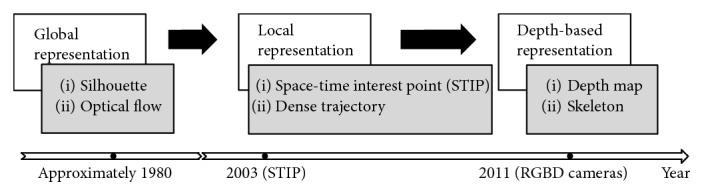
Research trajectory of activity representation approaches.

This review highlights the advances of image representation approaches and classification methods in vision-based activity recognition. Generally, for representation approaches, related literatures follow a research trajectory of global representations, local representations, and recent depth-based representations (Figure 1). Earlier studies attempted to model the whole images or silhouettes and represent human activities in a global manner. The approach in [5] is an example of global representation in which space-time shapes are generated as the image descriptors. Then, the emergency of space-time interest points (STIPs) proposed in [6] triggered significant attention to a new local representation view that focuses on the informative interest points. Meanwhile, local descriptors such as histogram of oriented gradients (HOG) and histogram of optical flow (HOF) oriented from object recognition are widely used or extended to 3D in HAR area. With the upgrades of camera devices, especially the launch of RGBD cameras in the year 2010, depth image-based representations have been a new research topic and have drawn growing concern in recent years.

On the other hand, classification techniques keep developing in step with machine learning methods. In fact, lots of classification methods were not originally designed for HAR. For instance, dynamic time warping (DTW) and hidden Markov model (HMM) were first used in speech recognition [7, 8], while the recent deep learning method is first developed for large amount image classification [9]. To measure these approaches with same criterion, lots of activity datasets are collected, forming public and transparent benchmarks for comparing different approaches.

In addition to the activity classification approaches, another critical research area within the HAR scope, the human tracking approach, is also reviewed briefly in a separate section. It is widely concerned especially in video surveillance systems for suspicious behavior detection.

The writing of rest parts conforms to general HAR process flow. First, research emphases and challenges of this domain are briefly illustrated in Section 2. Then, effective features need to be designed for the representation of activity images or videos. Thus, Sections 3 and 4, respectively, review the global and local representations in conventional RGB videos. Depth image-based representations are discussed as a separate part in Section 5. Next, Section 6 describes the classification approaches. To measure and compare different approaches, benchmark datasets act an important role on which various approaches are evaluated.  Section 7 collects recent human tracking methods of two dominant categories. In Section 8 we present representative datasets in different levels. Before we conclude this review and the future of HAR in Section 8, we classify existing literatures with a detailed taxonomy (Table 1) including representation and classification methods, as well as the used datasets aiming at a comprehensive and convenient overview for HAR researchers.

## 2. Challenges of the Domain

### 2.1. Intraclass Variation and Interclass Similarity

Different from speech recognition, there is no grammar and strict definition for human activities. This causes twofold confusions. On one hand, the same activity may vary from subject to subject, which leads to the intraclass variations. The performing speed and strength also increase the interclass gaps. On the other hand, different activities may express similar shapes (e.g., using a laptop and reading). This is termed as interclass similarity which is a common phenomenon in HAR. Accurate and distinctive features need to be designed and extracted from activity videos to deal with these problems.

### 2.2. Recognition under Real-World Settings

#### 2.2.1. Complex and Various Backgrounds

While applications like video surveillance and fall detection system use static cameras, more scenarios adopt dynamic recording devices. Sports event broadcast is a typical case of dynamic recording. In fact, with the popularity of smart devices such as smart glasses and smartphones, people tend to record videos with embedded cameras from wearable devices anytime. Most of these real-world videos have complex dynamic backgrounds. First, those videos, as well as the broadcasts, are recorded in various and changing backgrounds. Second, realistic videos abound with occlusions, illumination variance, and viewpoint changes, which make it harder to recognize activities in such complex and various conditions.

#### 2.2.2. Multisubject Interactions and Group Activities

Earlier research concentrated on low-level human activities such as jumping, running, and waving hands. One typical characteristic of these activities is having a single subject without any human-human or human-object interactions. However, in the real world, people tend to perform interactive activities with one or more persons and objects. An American football game is a good example of interaction and group activity where multiple players (i.e., human-human interaction) in a team protect the football (i.e., human-object interaction) jointly and compete with players in the other team. It is a challenging task to locate and track multiple subjects synchronously or recognize the whole human group activities as “playing football” instead of “running.”

#### 2.2.3. Long-Distance and Low-Quality Videos

**Figure 2:**
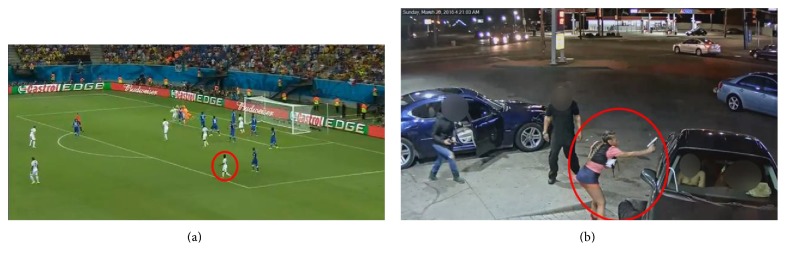
Long-distance videos under real-world settings. (a) HAR in long-distance broadcasts. (b) Abnormal behaviors in surveillance.

Long-distance and low-quality videos with severe occlusions exist in many scenarios of video surveillance. Large and crowded places like the metro and passenger terminal of the airport are representative occasions where occlusions happen frequently. Besides, surveillance cameras installed in high places cannot provide high-quality videos like present datasets in which the target person is clear and obvious. Though we do not expect to track everyone in these cases, some abnormal or crime-related behaviors should be recognized by the HAR system (Figure 2(b)). Another typical long-distance case is the football broadcast (Figure 2(a)). Due to the long distance of cameras, the subject is rather small which makes it difficult to analyze activities of the torso [10], and the relatively low quality of those long distance videos further increases the difficulty.

## 3. Global Representations

Global representations extract global descriptors directly from original videos or images and encode them as a whole feature. In this representation, the human subject is localized and isolated using background subtraction methods forming the silhouettes or shapes (i.e., region of interest (ROI)). Some global approaches encode ROI from which they derive corners, edges, or optical flow as descriptors. Other silhouette-based global representation methods stack the silhouette image along the time axis to form the 3D space-time volumes, then the volumes are utilized for representation. Besides, discrete Fourier transform (DFT) takes advantage of frequency domain information of ROI for recognition, also being a global approach. Global representation approaches were mostly proposed in earlier works and gradually outdated due to the sensitiveness to noise, occlusions, and viewpoint changes.

### 3.1. 2D Silhouettes and Shapes

To recognize the human activities in videos, an intuitive idea is to isolate the human body from the background. This procedure is called background subtraction or foreground extraction. The extracted foreground in the HAR is called silhouette, which is the region of interest and represented as a whole object in the global representation approach.

Calculating the background model is an important step before extracting silhouettes. Wren et al. [11] first proposed to model the background scene with Gaussian distribution. Koller et al. [12] pointed out that some foreground values update unduly and thus they introduced the selective background update strategy. Stauffer and Grimson [13] proposed to model the values of a particular background pixel as a mixture of Gaussians to replace the strategy of using only one Gaussian value in the previous approach. The Gaussian mixture model (GMM) has been applied widely but the introduction of expectation maximization (EM) algorithm increases the computational cost. To reduce the cost, *k*-means clustering algorithm is used to replace the EM algorithm with an insignificant loss of accuracy. It is worth mentioning that current RGBD cameras make it easy to obtain the silhouette by using the depth data provided by depth sensors.

Besides the silhouette representation, the 2D shape of the silhouette can be used as a feature as well. Veeraraghavan et al. [14] emphasized the effectiveness of shape features. In their experiments, shape and kinematics that are being considered as two important cues in human motion were evaluated. Tests on both the gait-based human identification and the activity recognition indicate that shape plays a more important role. Veeraraghavan et al. then used this shape representation in their following work [15].

Bobick and Davis [16, 17] stacked the silhouettes as two components for recognizing activities, respectively, the motion-energy image (MEI) and the motion-history image (MHI), which are both 2D representations.

In [18], oriented rectangular patches are extracted over the silhouettes. Spatial oriented histograms are then formed to represent the distribution of these rectangular patches. Those descriptors are finally used to recognize activities.

Extracting silhouettes from a single view is hard to satisfy view invariant property. To alleviate the influence of viewpoint changes, multiple cameras can be used to extract silhouettes in different viewpoints. Xu and Huang [19] proposed an “envelop shape” representation using two orthogonally placed cameras, which is robust to view changes of yaw rotation. Weinland et al. [20] made the same assumption that only the variations in viewpoints around the central vertical axis of the human body need to be considered. Motion history volumes (MHVs) were derived by stacking 4D silhouettes from four orthogonal cameras. In [21], a data fusion method was proposed, calculating the minimum DTW score between the test template and the two orthogonal view training templates.

### 3.2. Optical Flow

Optical flow is an effective way to extract and describe silhouettes for a dynamic background. Lucas-Kanade-Tomasi (LKT) feature tracker [22, 23] can be used to obtain the optical flow. Lu et al. [24] used a LKT feature tracker approach to track joints in key frames and actual frames. Each activity is represented as a posture sequence, and each key posture is recorded in a key frame. Specific posture in actual frames can be recognized by finding correspondence between the actual and key frame. The recognized posture from the actual frame is compared to the key posture frame by mapping body locations, and the matched posture sequences are confirmed as the activity.

For recognizing human activities at a distance (i.e., the football broadcast video), Efros et al. [10] introduced a descriptor based on computing the optical flow to describe the “small” football players in person-centered images. Obviously, the background is dynamic due to the movement of players which makes it hard to model for background subtraction.

Tran and Sorokin [25] combined silhouettes and optical flow features together. Normalized bounding box is scaled to capture the region of the human body, and the optical flow measurements within the box are split into horizontal and vertical channels, while the silhouette gives the third channel. Subwindows are further divided to calculate histograms, and concatenating histograms of all 3 channels form the final descriptor.

### 3.3. 3D Space-Time Volumes (STVs)

An activity video can be seen as a series of images that contain activity sequences. Concatenating all frames along the time axis forms the 3D space-time volume (STV) which has three dimensions including two spatial dimensions *X* and *Y* and one temporal dimension *T*. Representations based on STVs expect to capture the additional dynamic information which the spatial representation methods cannot obtain due to the absence of time dimension. Constructing STVs for different activities is a global representation method. However, the STV sometimes combines with local features to build the final feature sets.

Blank et al. [5] first introduced the space-time shape to represent human activities. Space-time shape is obtained by only stacking the silhouette regions within images. However, due to the nonrigidity of the constructed 3D space-time shapes and inherent difference between space and time dimensions, traditional 3D shape analysis cannot be applied to the space-time activity shapes. Thus, the solution of the Poisson equation is used to derive local space-time saliency and orientation features.

Achard et al. [26] generated semiglobal features named space-time micro volumes from image sequence to deal with performances of different temporal durations. Motivated by seeking the common underlying induced motion fields of sequences of the same behaviors, Shechtman et al. [27] proposed an approach to compare volumes according to their patches. This method requires no prior modeling or learning of activities, being able to handle the complex dynamic scenes and detect multiple activities that occur simultaneously within the camera view. Their method is partially invariant to the changes in scale and orientation.

In [28], the input videos are segmented into space-time volumes using mean shift clustering technique. These oversegmented regions, which are termed “super-voxels,” are then matched using a proposed shape-matching technique, which is compared to the traditional silhouette matching methods. Unlike the previous silhouette-based approaches, the proposed shape-based representation does not require background subtraction nor explicit background models. To avoid the shortages of the shape-matching methods that are ignoring features inside the shape, Shechtman and Irani's flow-based features [27] are further incorporated.

### 3.4. Discrete Fourier Transform (DFT)

The DFT of image frame is another global feature that contains the intensity information of the foreground object (i.e., the region of the subject's body) provided that the foreground object intensity is different from the background. Kumari and Mitra [29] took advantage of this hypothesis and proposed a DFT-based approach, obtaining information about the geometric structure of the spatial domain foreground object. Normalized image frame is divided into small size blocks within which the average of all the DFT values is calculated. Finally the K-nearest neighbor (KNN) is applied to classify the DFT features and generate the activity classification result. The extracted DFT feature is novel compared to the previous work; however, its performance is restricted to simple backgrounds. The background in their test video datasets is almost blank.

## 4. Local Representations

Instead of extracting the silhouette or STV and encoding them as a whole, local representations process activity video as a collection of local descriptors. They focus on specific local patches which are determined by interest point detectors or densely sampling [30]. Most existing local features are proved to be robust against noise and partial occlusions comparing to global features. Local features are then normally combined with the bag-of-visual-words (BoVW) model and yield the general pipeline of current state-of-the-art local representation approaches [31]. Oriented from bag-of-words (BoW), BoVW-based local representation mainly contains four steps: feature extraction, codebook generation, feature encoding, and pooling and normalization. We follow [32] and state a traditional BoVW pipeline here: interest points and local patches are first obtained by detectors or densely sampled. Then local features are extracted from those interest points or patches. Next, a visual dictionary (i.e., codebook) is learned in training set by *k*-means or Gaussian mixture model (GMM), the original high-dimension descriptors are clustered, and the center of each cluster is regarded as a visual codeword. After that, local features are encoded and pooled. Finally, the pooled vectors are normalized as video representation. Among these steps, the development of more elaborately designed low-level features and more sophisticated encoding methods are the two chief reasons for the great achievements in this field [32, 33], so in this part, we review the feature extraction methods in Section 4.1 and Section 4.2, as well as the encoding methods in Section 4.3.

### 4.1. Spatiotemporal Interest Point Detector

An intuitive thought of local representation is to identify those interest points that contain high information contents in images or videos. Harris and Stephens [34] first proposed effective 2D interest point detectors, the well-known Harris corner detector, which is extensively used in object detection. Then, Laptev and Lindeberg [6] proposed the 3D space-time interest points (STIPs) by extending Harris detectors. Spatial interest points in images are extended to spatiotemporal local structures in videos where the image values have significant local variations in both space and time. The spatiotemporal extents of the detected points are estimated by maximizing a normalized spatiotemporal Laplacian operator over spatial and temporal scales.

Saliency can also be used to detect interest points. Saliency means that certain parts of an image are preattentively distinctive and are immediately perceivable [35]. The spatiotemporal salient point can be regarded as an instance of the spatiotemporal interest point since both of them are informative and contain significant variations. The 2D salient point detection was first proposed by Kadir and Brady in [35]. Oikonomopoulos et al. [36] extended the 2D saliency to 3D spatiotemporal salient points that are salient both in space and time field. The salient points are successfully used as local features in their proposed activity classification scheme. Blank et al. [5] used the solution to Poisson equation to extract local space-time saliency of moving parts in the space-time shape. The detected salient points along with the local orientation and aspect ratios of shapes are calculated as local features.

Although these methods achieved remarkable results in HAR, one common deficiency is the inadequate number of stable interest points. In fact, the trade-off between the stability of those points and the number of points found is difficult to control. On one hand, the “right” and “discriminative” (i.e., stable) interest points are rare and difficult to be identified. As stated in [37], the direct 3D counterparts to commonly used 2D interest point detectors are inadequate, and true spatiotemporal corners are quite rare in certain applications. On the other hand, false alarms occur frequently due to various factors such as unintentional appearance changes. Ke et al. [38] illustrated two instances to point out that original detectors may fail in situations where the motions contain no sharp extrema; however, these detectors can be triggered falsely by the appearance of shadows and highlights in video sequences.

Besides the inherent properties of sparse interest points, many of the mentioned methods are inefficient. Therefore, these methods are restricted to the detection of a small number of points, or limited to low-resolution videos [39]. Here, we introduce some works either efficiency-enhanced or increasing number of stable interest points in response to the mentioned deficiency.

Dollar et al. [37] observed the rarity of the spatiotemporal interest points and the consequent problems of it in the recognition scheme. To find more 3D interest points in cuboids of space and time for activity recognition, the response function calculated by the separable linear filters is applied. The filtering is applied separately on the spatial and temporal dimensions, that is, 2D Gaussian smoothing kernel applied in spatial dimensions, and 1D Gabor filters applied in temporal dimension. Number of interest points increases using their detectors. Ke et al. [38] doubted the assumption that one can reliably detect a sufficient number of stable interest points in the video sequence. They extended the notion of rectangle features [40] into spatiotemporal volumetric features and applied the proposed framework on the video's optical flow. Their classifier is not limited to the sparseness nor affected by the instability of detected points.

Aiming at detecting interest points in an efficient way, Willems et al. [39] presented a dense, scale-invariant yet efficient spatiotemporal interest point detector with minimal effect on the computation time. First, point localization and scale selection are combined in a direct way using the determinant of the 3D Hessian matrix, therefore removing the time-consuming iterative scheme [41]. Further, building on Ke et al.'s work [38], an implementation scheme using integral video is developed to compute scale-invariant spatiotemporal features efficiently. Using a completely different idea, Oshin et al. [42] proposed to learn a classifier capable of detecting interest points in a novel video, given examples of the type of interest point that wish to get within a training video. The spatiotemporal Fern classifier (i.e., a seminaïve Bayesian classifier in [43]) is trained to recognize spatiotemporal interest points and thus achieves a high efficiency in constant time regardless of original detector complexity.

### 4.2. Local Descriptors

Local descriptors are designed to describe the patches that sampled either densely or at the interest points [1]. Effective descriptors are considered to be discriminative for the target human activity events in videos and robust to occlusion, rotation, and background noise.

Laptev [41] represented their 3D Harris corner by computing local, spatiotemporal N-jets as the descriptor. The descriptor is scale-invariant since they estimate the spatiotemporal extents of detected events by maximizing a normalized spatiotemporal Laplacian operator over spatial and temporal scales. Moreover, the proposed descriptors are proved to be robust to occlusions and dynamic cluttered backgrounds in the human motion analysis.

Similar to works of extending 2D interest point detector into spatiotemporal domain, such as the Harris corner detector [34] and the extended spatiotemporal one [41], many spatiotemporal descriptors were proposed by extending mutual image descriptors as well. We briefly review these works including both the original spatial descriptors and the spatiotemporal version of them.

Lowe proposed the scale-invariant feature transform (SIFT) in 1999 [44] and further improved it in 2004 [45]. It is widely used in local representation due to its scale and rotation invariance, as well as the robustness to affine distortion, changes in 3D viewpoint, addition of noise, and change in illumination. Scovanner et al. [46] introduced a 3D SIFT descriptor and used it in HAR. The 2D gradient magnitude and orientation are extended in 3D formulation; thus, creating the subhistograms encode the 3D SIFT descriptor. The videos are then described as a bag of spatiotemporal words using the 3D SIFT descriptor. Moreover, a feature grouping histogram which groups the co-occurred words out of the original one is used to build a more discriminative action video representation and finally used for classification.

The speeded-up robust features (SURF) [47] approach is a scale and rotation invariant detector and descriptor. The most important property of SURF is the improvement of efficiency comparing to previous approach. In the interest point detection, the approach applies the strategy that analyzing the input image at different scales to guarantee invariance to scale changes. Taking computation time into account, a very basic Hessian-matrix approximation which lends itself to the use of integral images is used for interest point detection, and it reduced the computation time dramatically. Next, a rotation and scale-invariant descriptor is provided for the detected interest point. The SURF approach builds on the distribution of first-order Haar-wavelet responses within the interest point neighborhood, in contrast with SIFT that extracts gradient information. Furthermore, integral images are exploited for speed. The introduction of indexing step based on the sign of the Laplacian further increases the robustness of descriptor and the matching speed.

An extended 3D SURF descriptor was implemented by Willems et al. [39]. Both of the 2D and 3D SURF used Haar-wavelet responses; however, the 3D SURF store the vector of the 3 axis responses instead of including the sums over the absolute values since the latter proved to be of no significant benefit but doubling the descriptor size.

Dalal and Triggs [48] proposed the histogram of oriented gradients (HOG) descriptor and achieved great success in human detection with linear SVM classifier. The good performance is due to the fact that the HOG's density distribution of local intensity gradients or edge directions can well characterize the local object appearance and shape of target objects.

Lu and Little et al. [49] presented the PCA-HOG descriptor which projects the original histogram of oriented gradients (HOG) descriptor to a linear subspace by principle component analysis (PCA). The descriptor was used to represent athletes to solve the problem of tracking and activity recognition simultaneously. Using HOG and HOF (histogram of flow) descriptor, Laptev et al. [50] completes a similar but more challenging activity recognition task as those activities are extracted from movies.

Klaser et al. [30] generalized the HOG descriptor to video sequences and proposed the HOG3D. Integral images are extended to integral videos for efficient 3D gradient computation. Polyhedrons are utilized for orientation quantization as an analogy of polygons in 2D space HOG. Optimized parameters for activity recognition have also been explored in their work.

Early spatiotemporal methods adopt a perspective of regarding the video as *x*-*y*-*t* 3D volumes [30, 39, 46]. However, recent feature trajectory approach considers the spatial dimensions *x*-*y* very different from the temporal dimension *t*. This approach detects the *x*-*y* interest points from video frames and then tracking them through video sequences as a trajectory. For detecting interest point, classic 2D detectors such as HOG and HOF are still used. In this review, we treat the feature trajectory as a special kind of the spatiotemporal descriptors where the time dimension is used to concatenate those 2D interest points.

Wang et al. [51] proposed dense trajectories by densely sampling points. Avoiding extracting points frame by frame and concatenating them, Wang et al. firstly extracted dense optical flow using Farneback's algorithm [52], then points can be densely tracked along the trajectory without additional cost. HOG and HOF are computed along the dense trajectories as the descriptors. Dense trajectories were further improved in [53]. The camera motion, as a main obstacle for extracting target trajectories from humans or objects of interests, was highlighted and was tried to be removed. The authors first match feature points using two complementary descriptors (i.e., SURF and dense optical flow), then estimate the homography using RANSC [54]. Through this approach, the camera motion is explicitly identified and removed. However, in some cases where humans dominate the frame, the target human motion may also generate inconsistent camera motion match. To solve this problem, a human detector is further explored to remove the inconsistent matches within the detected human areas. Improved descriptors achieved significant performance on challenge datasets, such as Hollywood2 where camera motions were used abundantly. Shi et al. [55] presented a sequential deep trajectory descriptor (sDTD) on the dense trajectory basis to capture the long-term motion information. The dense trajectories are projected into two-dimensional planes and a CNN-RNN network is employed to learn an effective representation for long-term motion.

### 4.3. Feature Encoding Methods

The STIP-based descriptors or other elaborately designed descriptors are all referred as local features. Local features are then encoded with feature encoding methods to represent activities and the encoded features are subsequently fed into pretrained classifiers (e.g., SVM) [32]. Encoding feature is a key step for constructing BoVW representation and utilizing an appropriate encoding method can significantly improve the recognition accuracy [56]. Here, we summarize the common feature encoding methods in recent literatures in Table 2. The number of citations for each description paper is also provided to facilitate measurement of their influences.

Several evaluations [56–58] have been conducted to compare the performance of recent encoding methods. Chatfield et al. [57] compared five encoding methods including LLC, SVC, FV, KCB, and the standard spatial histograms baseline. Experiments over PASCAL VOC 2007 and Caltech 101 show that FV performs best. Wang et al. [56] drew the same conclusion on KTH dataset and HMDB51 dataset. Also, a most recent evaluation [58] showed a consistent finding on UCF-YouTube and HMDB51 datasets, though slightly slower than local NBNN on KTH.

Further exploration has been conducted to match the best local feature with FV. In [31], six representative methods including VQ, SA-*k*, LLC, FV, VLAD, and SVC are evaluated for two widely used local features, STIPs and improved dense trajectories (iDTs). The experiment results demonstrate that the iDT together with the FV yields the best performance on the test datasets. Wang et al. who proposed the iDT also verified the best performance of iDT and FV in their work [53].

**Figure 3:**
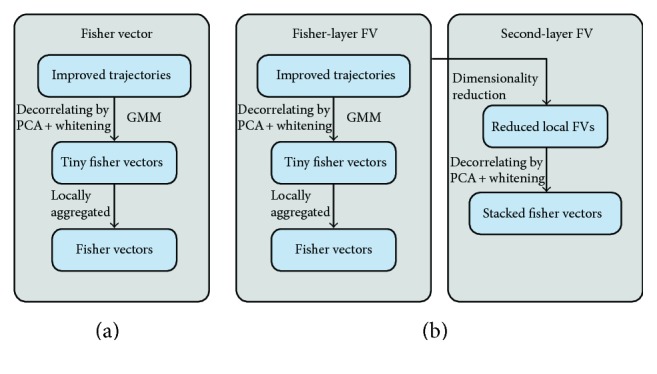
Pipeline of Fisher vector and Stacked fisher vector. (a) Fisher vector. (b) Stacked fisher vector.

Recent stacked Fisher vectors [32] further improved the performance of iDT + FV and achieved superior performance when combining traditional FV. Evaluation on the YouTube, J-HMDB, and HMDB51 datasets demonstrates that it has become the state-of-the-art method. Pipelines of SFV and corresponding FV are given in Figure 3.

The core idea of both FV and SFV is trying to catch more statistical information from images; in contrast, BoVW only retains the zero order statistics. Take an *l*-dimension local descriptor as an example. Assuming that the size of prelearned GMM is *K* (*K* is the size of codebook). For the conventional BoVW, the final encoded feature is *K*-dimension histograms that indicate the frequency of codewords. However, FV can obtain a 2*Kd*-dimension (*d* is the Gaussian distribution dimension). In another word, FV retained more information (i.e., high-order statistics) regarding to same size of codebooks.

SFV further improved FV owing to a simple and intuitive reason that SFV densely calculated local features by dividing and scanning multiscale subvolumes. The main challenge is the holistic combination of those local FVs since encoding them using another FV directly is impossible because of the high dimension of them (2*Kd*-dimension). Thus, a max-margin method is tactfully used to reduce dimensionality. As the local FVs are more densely sampled than the conventional FV and consequently contain more high order statistics, therefore, iDT with SFV achieves even better result than the state-of-the-art iDT with FV.

## 5. Depth-Based Representations

**Figure 4:**
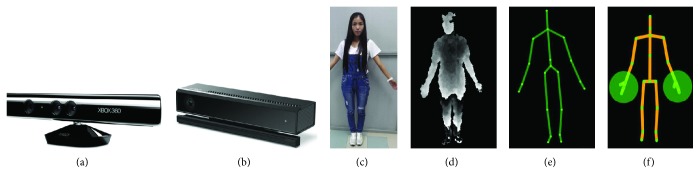
Kinect RGBD cameras and their color images, depth maps, skeletal information. (a) Kinect v1 (2011). (b) Kinect v2 (2014). (c) Color image. (d) Depth map. (e) Skeleton captured by Kinect v1. (f) Skeleton captured by Kinect v2.

Previous research of HAR mainly concentrates on the video sequences captured by traditional RGB cameras. Depth cameras, however, have been limited due to their high cost and complexity of operation [74]. Thanks to the development of low-cost depth sensors such as Microsoft Kinect [75], an affordable and easier way to access the depth maps is provided. Furthermore, Kinect SDK released the application that can directly obtain the skeletal joint positions in real-time (adopting algorithms in [75]). The available depth maps and the skeletal information (see Figure 4) vigorously contributed to the computer vision community. These two features and their derivative features also triggered a wide interest to solve HAR problems using depth-based solutions, replacing conventional RGB-based methods, or acting as supplements to enhance the RGB-based methods. In this section, we separately reviewed the recent advance of activity representations using depth maps or skeletons.

### 5.1. Representations Based on Depth Maps

Depth maps contain additional depth coordinates comparing to conventional color images and are more informative. Approaches presented in this section regard depth maps as spatiotemporal signals and extract features directly from them. These features are either used independently or combined with RGB channel to form multimodal features.

Li et al. [76] employed the action graph model, which represents activities using several salient postures serving as nodes in action graph. All activities share same posture sets and each posture is characterized as a bag of 3D points from the depth maps. However, involving all the 3D points is computationally expensive; thus, a simple and effective method to sample the representative 3D points is proposed, achieving over 90% recognition accuracy by sampling approximately 1% points according to their report.

Zhao et al. [77] proposed a framework of combing RGB and depth map features for HAR and presented an optimal scheme. For the RGB channels, spatiotemporal interest points are generated solely from it and the HOG and HOF are calculated to form the RGB based descriptors. For the depth channel, they proposed a depth map-based descriptor called local depth pattern (LDP), which simply calculates the difference of average depth values between a pair of cells within the STIP surrounding region.

Yang et al. [78] proposed to use HOG on depth maps. Depth maps are projected onto three orthogonal planes and the depth motion maps (DMM) are generated by accumulating global activities through entire video sequences. HOG are then computed from DMM as the representation of an action video. Another depth image-based work similar to the HOG is [74] where the histogram of oriented 4D normals (HON4D) descriptor, as a further generalization of HOG3D to four-dimensional depth videos, is proposed. HON4D descriptor calculates the histograms of oriented 4D surface normals in 4D space of time, depth, and spatial coordinates. A quantization of the 4D space is also presented. The approach in [79] is also based on the polynormal which is a cluster of neighboring hypersurface normals from a local spatiotemporal depth volume. A designed scheme aggregates the low-level polynormals in each adaptive spatiotemporal cell. The concatenation of feature vectors extracted from all spatiotemporal cells forms the final representation of depth sequences.

Jalal et al. [80] considered multifeatures from depth videos, extracting 3D human silhouettes and spatiotemporal joints values for their compact and sufficient information for HAR task.

### 5.2. Skeleton-Based Representations

Skeletons and joint positions are features generated from depth maps. Kinect device is popular in this representation due to its convenience of obtaining skeleton and joints. Application in Kinect v1 SDK generates 20 joints, while the later version (Kinect v2) generates 25 joints, adding 5 joints around the hands and neck (see Figure 4). We reviewed recent papers on skeleton-based representations and summarize three aspects efforts on improving the performance of skeleton-based representation.

**Figure 5:**
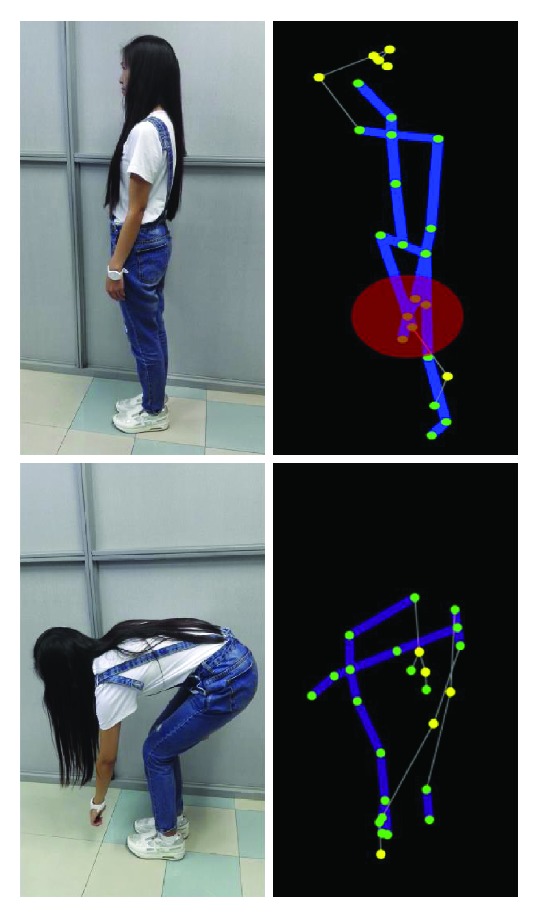
Noisy skeleton problem caused by self-conclusion.

First, skeleton model has an inherent deficiency that it always suffers the noisy skeleton problem when dealing with occlusions (see Figure 5) [76]. Features from inaccurate skeletons and joints may completely be wrong. Current approaches often solve it by combining other features that robust to occlusion or alleviate occlusion problem by separating the whole skeleton into different body parts and handling them independently since not all body parts are occluded.

Second, an intuitive fact can be observed that not all skeletal joints are involved in a particular activity, and only a few active joints are meaningful and informative for a certain activity [81]. Concentrating on these active joints and abandoning the other inactive parts will generate more discriminative and robust features and are beneficial to deal with intraclass variations [82].

Finally, as an extracted feature from depth maps itself, skeleton-based representation is often combined with original depth information to form more informative and robust representation [82, 83].

Xia et al. [84] proposed a skeleton-based representation named HOJ3D, the spherical histograms of 3D locations of selected joints. After reprojected using LDA and clustered into vocabularies, the encoded features are fed to hidden Markov model (HMM) for classification. The HOJ3D is robust to view changes due to the design of the spherical coordinate system and robust skeleton estimation.

Yang and Tian [85] proposed a new type of feature named EigenJoints. 3D position differences of joints are employed to characterize three kinds of activity information including posture feature, motion feature, and offset feature. To reduce redundancy and noise, PCA is further employed and the efficient leading eigenvectors are selected. Finally, the constructed features were fed into the naïve-Bayes-nearest-neighbor (NBNN) [86] and obtained improved performance.

Wang et al. [82] indicated that using joint positions alone is insufficient to represent an action, especially for the case involving interaction with objects. Consequently, they proposed a depth-based feature called local occupancy pattern (LOP) to describe the occupancy of the neighborhood of each point, for example, the occupied space around the hand joint when lifting a cup. The local occupancy information is described by the 3D point cloud around a particular joint. Moreover, to select the active and discriminative joint feature subset (i.e., actionlet) for a particular activity, a data mining solution is leveraged and then actionlet ensemble which is linear combination of actionlets is obtained to represent each activity. Similar to actionlet, Zhu et al. [87] learned the co-occurrences of joints by designing regularization in deep LSTM (long short-term memory) RNNs (recurrent neural networks).

Shahroudy et al. [83] proposed a multimodal multipart approach for activity recognition in depth map sequences, which combines the complementary skeleton-based features LOP in [82] and depth-based features local HON4D in [74] of each part together and builds up a multimodal multipart combination. The multimodal multipart features are formulated into their framework via the proposed hierarchical mixed norm.

Chen et al. [81] proposed a skeleton-based two-level hierarchical framework. In the first layer, a part-based clustering feature vector is introduced to find out the most relevant joints and clustered them to form an initial classification. Note that the recognition task is divided into several smaller and simple tasks, which are performed within a specific cluster. It is of benefit to solving the high intraclass variance since distinct sequences of the same action are grouped into different clusters. In the second layer, only the relevant joints within specific clusters are utilized for feature extraction, which enhances the validity of the features and reduces the computational costs.

Besides depth-based features, skeleton data can be combined with other RGB features. To deal with the noisy skeleton problem, Chaaraoui et al. [88] proposed to combine skeletal and silhouette-based features using feature fusion methods. The noisy skeleton problem caused by occlusions of body part is partially elevated by the silhouette-based features. Shahroudy et al. [83] separately extracted dense trajectories features from RGB channel and 3D locations of skeleton joints from depth channel. A hierarchical feature fusion method based on structured sparsity was developed to fuse these two heterogeneous features.

## 6. Activity Classification Approaches

The next stage of HAR is the classification of activities that have been represented by proper feature sets extracted from images or videos. In this stage, classification algorithms give the activity label as final result. Generally speaking, most activity classification algorithms can be divided into three categories namely template-based approaches, generative models and discriminative models. Template-based approaches is a relatively simple and well accepted approach; however, it can be sometimes computationally expensive. Generative models learn a model of the joint probability *P(X,Y)* of the inputs *X* and the label *Y*, then *P(Y|X)* is calculated using Bayes rules and the algorithms finally picking the most likely label *Y* [89]. In contrast, discriminative models determine the result label directly. Typical algorithms of generative models are hidden Markov model (HMM) and dynamic Bayesian network (DBN), while support vector machine (SVM), relevance vector machine (RVM), and artificial neural network (ANN) are typical discriminative models.

### 6.1. Template-Based Approaches

Template-based approaches try to portray common appearance characteristics of a certain activity using various representations. These common appearance characteristics, such as 2D/3D static images/volumes or a sequence of view models, are termed as templates. Most template-based methods extract 2D/3D static templates and compare the similarity between the extracted images/volumes of test videos and the stored templates. For the classification based on a sequence of key frames, dynamic time warping (DTW) is an effective approach.

#### 6.1.1. Template Matching

Bobick and Davis [16, 17] proposed a temporal-template-based approach. Two components, the motion-energy image (MEI) which represents the presence of motion and the motion-history image (MHI) which indicates the recency of motion, are generated for each template of an activity. In fact, the generated template images can be regarded as weighted projection of the space-time shape.

Shechtman and Irani [27, 90] constructed the 3D space–time intensity video volume template from a short training video clip. This small template is compared to every segment of same size in the test video over all three dimensions. The degree of similarity between two segments (i.e., the template and a same size video segment from the test video) is evaluated by the proposed intensity patch-based approach. It divides the segments into smaller patch units, then computes and integrates local consistency measures between those small space-time patches. This method has an impressive ability of detecting multiple different activities that occur at the same time.

Common template-based methods are unable to generate single template for each activity. They often suffer the high computational cost due to maintaining and comparing various templates. Rodriguez et al. [91] proposed to use the maximum average correlation height (MACH), which is capable of capturing intraclass variability by synthesizing a single action MACH filter for each activity class. They also generalized the MACH filter to video and vector valued data by embedding the spectral domain into a domain of Clifford algebras, building an effective approach in discriminating activities.

#### 6.1.2. Dynamic Time Warping

Dynamic time warping (DTW) is a kind of dynamic programming algorithm for matching two sequences with variances. Rabiner and Juang [7] first developed it for speech recognition problem, representing the words as template sequence and assign matching scores for new word. DTW is also applicable to HAR problem since the human activities can be viewed as a sequence of key frames. The recognition problem is transformed to a template matching task.

Darrell and Pentland [92] proposed to build the representation of gestures using a set of learned view models. DTW algorithm is used to match the gesture template obtained from the means and variations of correlation scores between image frames and view models.

Veeraraghavan et al. [93] proposed the DTW-based nonparametric models for the gait pattern problem. They modified the DTW algorithm to include the nature of the non-Euclidean space in which the shape deformations take place. By comparing the DTW-based nonparametric and the parametric methods and applying them to the problem of gait and activity recognition, this work concluded that the DTW is more applicable than parametric modeling when there is very little domain knowledge.

Although the DTW algorithm needs a few amounts of training samples, the computational complexity increases significantly when dealing with growing activity types or those activities with high inter/intra variance, because extensive templates are needed to store those invariance.

### 6.2. Generative Models

#### 6.2.1. Hidden Markov Model Approach

The recognition task is a typical evaluation problem which is one of the three hidden Markov model problems and can be solved by the forward algorithm. HMMs were initially proposed to solve the speech recognition problem [8]. Yamato et al. [94] first applied the HMM to recognize activities. Features that indicate the number of pixels in each divided mesh are obtained as observations for each frame. Then, the HMMs are trained using the observation feature vector sequences for each activity, including the initial probability of hidden states, the confusion matrix, and the transition matrix. By applying the representation mentioned above, the HAR problem (recognition of various tennis strokes) is transformed into a typical HMM evaluation problem, which can be solved using standard algorithm.

A brief summary of the deficiencies of basic HMM and several efficient extensions are presented in [95]. The basic HMM is ill-suited for modeling multiple interacting agents or body parts since it is single variable state representation, as well as those actions that have inherent hierarchical structure. Take human interaction as an example, as a kind of complex activities, it always contains more than one person in the video, to which the basic HMM is ill-suited since the standard HMM is suitable for the time structure. Another deficiency is the exponentially decayed duration model for state occupancy. This duration model has no memory of the time that has already spent on the state, which is unrealistic for activities. This is implicitly obtained from the constant state transition probability and the first-order Markov assumption, which implies that the probability of a state being observed for a certain interval of time decays exponentially with the length of the interval [96].

Previous work has proposed several variants of HMM to handle the mentioned deficiencies [95–97]. Motivated by this human interaction recognition task that have structure both in time and space (i.e., modeling activities of two or more persons), Oliver et al. [97] proposed the coupled HMM (CHMM) to model the interactions. Two HMM models are constructed for two agents and probabilities between hidden states are specified.

Flexible duration models were suggested including the hidden semi-Markov model (HSMM) and the variable transition HMMs (VT-HMM). The hidden semi-Markov model (HSMM) is a candidate approach that has explicit duration model with specific distribution. Duong et al. [98] exploited both the inherent hierarchical structure and the explicit duration model and the switching hidden semi-Markov model (S-HSMM) is introduced with two layers to represent high-level activities and atomic activities separately. Another semi-Markov model (HSMM) based work is shown in [96].

Alternatively, Ramesh and Wilpon [99] broke the implicit duration model by specifying the dependency between the transition probability and the duration. The variable transition HMMs (VT-HMMs, originally called inhomogeneous HMM in [99]) was proposed and applied in speech recognition. In VT-HMM, the transition probability of two states depends on the duration which is no longer constant. Natarajan and Nevatia [95] then presented a hierarchical variable transition HMM (HVT-HMM) based on Pamesh and Wilpon's work to recognize two-hand gestures and articulated motion of the entire body. The HVT-HMM has three layers, including a composite event layer with a single HMM representing the composite actions, a primitive event layer using a VT-HMM to represent the primitive actions, and a pose track layer with a single HMM. The pose is represented using a 23 degrees body model, including 19 degrees for joint angles, 3 degrees for direction of translation (*x*, *y*, *z*), and 1 degree for scale.

#### 6.2.2. Dynamic Bayesian Networks

A dynamic Bayesian network (DBN) is a Bayesian network with the same structure unrolled in the time axis [100]. An important extension of DBN is that its state space contains more than one random variables, in contrast with the HMM that has only one single random variable. Thus, the HMM can be viewed as a simplified DBN with constrained number of random variables and fixed graph structures.

**Figure 6:**
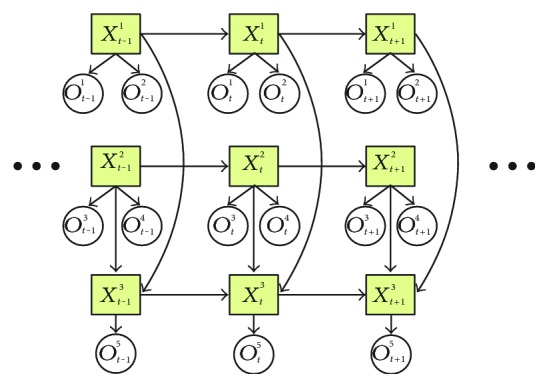
A typical dynamic Bayesian network [101].

Figure 6 presents a typical DBN. Suk et al. [101] proposed this structure for two hands gesture recognition, from which we can see that there are three hidden variables. The three hidden variables represent the motion of two hands and their spatial relation, while five features including two hands' motion and the position relative to the face, as well as the spatial relation between hands are designed as observations. Then, the DBN structure is built and simplified using the first-order Markov assumptions. They proposed the DBN tailored for hands gesture recognition in contrast with the previous fixed structure of CHMM [102] which is not deemed effective for other than tight-coupled two-party interactions.

Park and Aggarwal [103] presented a hierarchical Bayesian network methodology for recognizing five two-person interactions. The proposed method first segments the body-part regions and estimates each of the body-part poses separately in the first level. Then, the individual Bayesian networks are integrated in a hierarchy to estimate the overall body poses of a person in each frame. Finally, the pose estimation results that include two-person interactions are concatenated to form a sequence with DBN algorithm.

Cherla et al. [21] indicated the contradiction for DTW between the robustness to intraclass variations and the computational complexity. Multiple templates for each activity handle the intraclass variations well but increase the computational complexity, while average templates reduce the complexity but are sensitive to intraclass variations. Cherla et al. proposed the average template with multiple feature representations to counterbalance them and achieve good performance.

### 6.3. Discriminative Models

#### 6.3.1. Support Vector Machines

Support vector machines (SVMs) are typical classifiers of discriminative models and gained extensive use in HAR. Vapnik et al. [104] designed the SVM and originally used it for the problem of separating instances into two classes. It aims to find the hyperplane which maximizes the margin of two classes.

Schüldt et al. [105] combined SVM with their proposed local space-time features and applied their “local SVM approach” for HAR. A video dataset, known as the KTH dataset which had been one of the benchmarks of HAR systems, was recorded by them. The KTH dataset is introduced later in this paper (see Section 8.2.1).

Laptev et al. [50] used a nonlinear SVM with a multichannel Gaussian kernel and their SVM achieved high accuracy (91.8%) on the KTH dataset along with the HOG&HOF descriptors and local spatiotemporal bag-of-features. The well-known challenging Hollywood dataset (see Section 8.3.1) was provided and used to evaluate the proposed approach.

#### 6.3.2. Conditional Random Fields

Conditional random fields (CRFs) are undirected graphical models that compactly represent the conditional probability of a particular label sequence *Y*, given a sequence of observations *X*. Vail et al. [106] compared the HMMs and CRFs for activity recognition. They found that the discriminatively trained CRF performed as well as or better than an HMM even when the model features are in accord with the independence assumptions of the HMM. This work pointed out a significant difference between the HMMs and CRFs: the HMMs assume that observations are independent given their labels; thus, complex features of the observation sequence will invalidate the assumption of this model and then make the HMM no longer a proper generative model. This inherent assumption of HMMs is abandoned in CRF, which conditions on the entire observation and therefore does not require any independence assumptions between the observation variables. A test was done by incorporating features which violate independence assumptions between observations (i.e., velocity thresholds in [106]) to explore the influence on both models. The result demonstrates that the CRF always outperforms the HMM, and with the increasingly severe violation of the independence assumptions, the HMM gets worse.

Natarajan and Nevatia [107] presented an approach for recognizing activities using CRF. Synthetic poses from multiple viewpoints are firstly rendered using Mocap data for known actions. Then, the poses are represented in a two-layer CRF, with observation potentials computed using shape similarity and transition potentials computed using optical flow. These basic potentials are enhanced with terms to represent spatial and temporal constraints, and the enhanced model is called the shape, flow, duration conditional random field (SFD-CRF). Single human activities as sitting down or standing up were recognized in their experiment.

Ning et al. [108] proposed a model that replaced the observation layer of a traditional random fields model with a latent pose estimator. The proposed model converted the high-dimensional observations into more compact and informative representations, and enabled transfer learning to utilize existing knowledge and data on image-to-pose relationship. This method has been shown to improve performance on the public available dataset HumanEva [109].

#### 6.3.3. Deep Learning Architectures

Basically, the deep learning architectures can be categorized into four groups, namely deep neural networks (DNNs), convolutional neural networks (ConvNets or CNNs), recurrent neural networks (RNNs), and some emergent architectures [110].

The ConvNets is the most widely used one among the mentioned deep learning architectures. Krizhevsky et al. [9] first trained the deep ConvNets in a sufficiently large image datasets consisting of over 15 million labeled images. The impressive results lead to the extensively used of ConvNets in various pattern recognition domains [111]. Compared with traditional machine learning method and their hand-crafted features, the ConvNets can learn some representational features automatically [112]. Mo et al. [113] used ConvNets directly for feature extraction, and a multilayer perceptron is designed for the following classification.

One challenge for HAR using deep learning is how to apply it on small datasets since HAR datasets are generally smaller than what the ConvNets need. Common solutions include generating or dumpling more training instances, or converting HAR to a still image classification problem to leverage the large image dataset (e.g., ImageNet) to pretrain the ConvNets. Wang et al. [114] developed three strategies to leverage ConvNets on small training datasets. First, 3D points of depth maps are rotated to mimic different viewpoints, and WHDMMs at different temporal scales are constructed. Second, ConvNets model trained over ImageNet is adopted through transfer learning. Finally, different motion patterns are encoded into the pseudo-RGB channels with enhancement before being input to the ConvNets. On the other hand, Simonyan and Zisserm [115] leverage the large image dataset to pretrain the ConvNets. They investigated an architecture based on two separate streams (spatial and temporal), while the spatial stream contains information on appearance from still frames and is implemented using a spatial stream ConvNet. The spatial ConvNet is image classification architecture itself; thus, it is pretrained on the large image classification dataset.

The most recent research aims to further improve the performance of ConvNets by combining it with other hand-crafted features or representations. Li et al. [116] noted that the long-range dynamics information is necessary and should be modeled explicitly. Thus, they proposed a representation named VLAD^3^, which not only captures short-term dynamics with ConvNets but also utilizes the linear dynamic systems and VLAD descriptor for medium-range and long-range dynamics. Wang et al. [117] proposed a trajectory-pooled deep-convolutional descriptor (TDD) which combined the hand-crafted local features (e.g., STIP, improved trajectories) and deep-learned features (e.g., 3D ConvNets [76, 118], two-stream ConvNets [115]). The proposed TDD integrates the advantages of these two features and adopts the state-of-the-art improved trajectories and two-stream ConvNets.

Unlike ConvNets, DNNs still use hand-crafted features instead of automatically learning features by deep networks from raw data. Berlin and John [119] used Harris corner-based interest points and histogram-based features as input. The proposed deep neural network with stacked auto encoders are used to recognize human-human interactions. Huang et al. [120] learned Lie group features (i.e., one of the skeletal data representations that are learned by manifold-based approaches) by incorporating a Lie group structure into a deep network architecture.

RNNs are designed for sequential information and have been explored successfully in speech recognition and natural language processing [121, 122]. Activity itself is a kind of time-series data and it is a natural thought to use RNNs for activity recognition.

Among various RNNs architectures, the long short-term memory (LSTM) is the most popular one as it is able to maintain observations in memory for extended periods of time [123]. As an initial study for activity recognition, a LSTM network was utilized to classify activities in soccer videos [124]. Then, further research [123] explicitly demonstrated the robustness of LSTM even as experimental conditions deteriorate and indicated its potential for robust real-world recognition. Veeriah et al. [125] extended the LSTM to differential recurrent neural networks (RNNs). By computing the different orders of derivative of state which is sensitive to the spatiotemporal structure, the salient spatiotemporal representations of actions are learned, while in contrast, the conventional LSTM does not capture salient dynamic patterns of activity.

In addition to videos, RNNs can also be applied to skeleton data for activity recognition. Du et al. [126] proposed a hierarchical RNNs structure for skeleton-based recognition. The human skeleton from Kinect are divided into five parts and are fed into subnets separately. Representations from subnets are hierarchically fused into a higher layer and finally fed into a single-layer perceptron, whose temporally accumulated output is the final decision.

A detailed taxonomy about the representation, classification methods, and the used datasets of the introduced works in this review are presented in Table 1.

## 7. Human Tracking Approaches

Besides the activity classification approaches, another critical research area is the human tracking approach, which is widely concerned in video surveillance systems for suspicious behavior detection. Human tracking is performed to locate a person along the video sequence over a time period, and then the resultant trajectories of people are further processed by expert surveillance systems for analyzing human behaviors and identifying potential unsafe or abnormal situations [127]. In this section, we briefly review recent literatures of two dominant approaches, namely kernel-based tracking and filtering-based tracking.

### 7.1. Filter-Based Tracking

Filtering is one of the widely used approaches for tracking, and the representative Kalman filter (KF) [128] and particle filter (PF) [129] are two commonly used classic filtering techniques.

KF is a state estimate method based on linear dynamical systems that are perturbed by Gaussian noise [130]. Patel and Thakore utilized traditional KF to track moving objects, in both the indoor and outdoor places. Vijay and Johnson [131] also utilized traditional KF for tracking moving objects such as car or human. However, the tested scenarios of these cases are relatively spacious and thus seldom occlusion occur. Despite the good results that are achieved by the KF-based method, it is strictly constrained with effective foreground segmentation, and its ability is limited when handling the occlusion cases. Arroyo et al. [127] combined Kalman filtering with a linear sum assignment problem (LSAP). To deal with the occlusion problem, visual appearance information is used with image descriptors of GCH (global color histogram), LBP (local binary pattern), and HOG (histogram of oriented gradients) representing the color, texture, and gradient information, respectively.

Particle filter, or sequential Monte Carlo method [132], is another typical filtering method for tracking. PF is a conditional density propagation method that is utilized to deal with non-Gaussian distributions and multimodality cases [130]. Ali et al. [133] combined a head detector and particle filter for tracking multiple people in high-density crowds. Zhou et al. [130] presented a spatiotemporal motion energy particle filter for human tracking, which fuses the local features of colour histograms as well as the spatiotemporal motion energy. The proposed particle filter-based tracker achieved robustness to illumination changes and temporal occlusions through using these features, as the motion energy contains the dynamic characteristics of the targeted human. As a specific branch of particle filter research, the sequential Monte Carlo implementation of the probability hypothesis density (PHD) filter, known as the particle PHD filter, is well developed for solving multiple human tracking problems. A series of research have been conducted by Feng et al. in [134–138].

### 7.2. Kernel-Based Tracking

Kernel-based tracking [139] or mean shift tracking [140] tracks the object (human) by computing the motion of one or more spatially weighted color histograms (i.e., single kernel/multiple kernels) from the current frame to next frame based on an iteratively mean-shift procedure. The kernel-based approach has fast convergence speed and low computation requirement inherited from the efficient mean shift procedure [141].

Traditional kernel-based tracking used symmetric constant kernel, and it tends to encounter problems of object scale and object orientation variation, as well as the object shape deformation. Research was conducted concerning these problems. Liu et al. [142] presented a kernel-based tracking algorithm based on eigenshape kernel. Yilmaz [143] introduced a kernel-based tracking algorithm based on asymmetric kernel for the first time. This kernel uses the initial region inside the outline of the target as kernel template and generates a precise tracking contour of the object. Yuan-ming et al. [144] noticed the shortage of the fixed asymmetric kernel. They combined the contour evolution technology with the mean shift and proposed an enhanced mean shift tracking algorithm based on evolutive asymmetric kernel. Liu et al. [145] presented an adaptive shape kernel-based mean shift tracker. Shape of the adaptive kernel is reconstructed from the low-dimensional shape space obtained by nonlinear manifold learning technique to the high-dimensional shape space, aiming to be adaptive to the object shape.

Early literatures reported tracking methods using single kernel scheme. However, the single kernel-based tracking could fail when the human is concluded, that is, the object could be lost or mismatch due to the partial observation. Thus, multiple-kernel tracking is adopted in most cases of recent researches. Lee et al. [146] evaluated two kernel and four kernel schemes [147] and presented a similar two and four kernal evaluation. Chu et al. [148] proposed to utilize projected gradient to facilitate multiple-kernel tracking in finding the best match under predefined constraints. The occlusion is managed by employing adaptive weights, that is, decreasing the importance of the kernel being occluded whilst enhancing the ones which are well-observed. Hou et al. [149] integrated the deformable part model (DPM) and designed multiple kernels, each of which corresponds to a part model of a DPM-detected human.

## 8. Representative Datasets in HAR

Public datasets could be used to compare different approaches in the same standards therefore accelerate the development of HAR methods. In this section, several representative datasets are reviewed, organized as a three-level category mentioned in the beginning of this review (i.e., action primitive level, action/activity level, and interaction level). There have been a published good survey [4] which presents the available important public datasets; however, it mainly focused on the conventional RGB-based datasets and missed current depth-based datasets. Thus, several important benchmark depth or RGB-D datasets are also reviewed in this section, with an overview of them (Table 3).

### 8.1. Action Primitive Level Datasets

While action primitives often act as components of high level human activities (e.g., the action primitives are served as a layer in hierarchical HMM to recognize activities [95] or interactions [97]), some typical and meaningful action primitives, such as poses and gestures [150], gait pattern [151], are studied as separate topics. These topics aroused wide research interest due to their importance in applications such as human-computer interaction and health care. Here, we present two recent gesture dataset based on RGB-D as the representative dataset in this level.

#### 8.1.1. NTU-MSR Kinect Hand Gesture Dataset (2013)

The NTU-MSR Kinect hand gesture dataset [152] is considered as an action primitive level since it is developed for gesture recognition. Gestures in it were collected by Kinect, and each of them consists of a color image and the corresponding depth map. Totally, 1000 cases of 10 gestures were collected by 10 subjects, and each gesture was performed 10 times by a single subject in different poses. The dataset is claimed as a challenging real-life dataset due to their cluttered backgrounds. Besides, for each gesture, the subject poses with variations in hand orientation, scale, articulation, and so forth.

#### 8.1.2. MSRC-Kinect Gesture Dataset (2012)

The MSRC-Kinect gesture dataset [153] is another typical action primitive level dataset, in which large amounts of limb level movements (e.g., karate kicking forwards with right leg) were recorded. There are totally 6244 instances of 12 gestures performed by 30 people, collected by Kinect. Positions of 20 tracked joints are provided as well.

### 8.2. Action/Activity Level Datasets

According to our definition, action/activity is middle level human activity without any human-human or human-object interactions. We first review two classic datasets, namely KTH human activity dataset and Weizmann human activity dataset. Though these two datasets have gradually faded out of state-of-the-art and are considered as easy tasks (e.g., 100% accuracy for Weizmann in [18, 25, 95]), they did play important roles in the history and act as benchmarks in earlier HAR works. Then, the well-known benchmark dataset for depth-based approaches, MSR Action3D dataset, is introduced next.

#### 8.2.1. KTH Activity Dataset (2004)

The KTH dataset [105] is one of the most frequently cited datasets. It contains 6 activities (walking, jogging, running, boxing, hand waving, and hand clapping) performed by 25 subjects in controlled sceneries including outdoors, outdoors with scale variation, outdoors with different clothes, and indoors. One important factor in their success is the high intraclass variation in it which is one of the criteria for evaluation algorithms. Although the videos were still taken using static cameras, the high variation details, such as various scenarios and actors' clothes, as well as the different viewpoints, make itself a fair and convincing datasets for comparison. Most of the collected human activities in it were performed by a single person without any human-object interaction; thus, it is categorized in the activity/action level.

#### 8.2.2. Weizmann Activity Dataset (2005)

The Weizmann activity dataset [5] was created by the Weizmann Institute of Science (Israel) in 2005. The Weizmann dataset consists of 10 natural actions (running, walking, skipping, bending, jumping-jack, galloping-sideways, jumping-forward-on-two-legs, jumping-in-place-on-two-legs, waving-two-hands, and waving-one-hand) with 10 subjects. Totally, 90 video sequences in a low resolution of 180^∗^144, 50 fps were recorded using a fixed camera and a simple background. To address the robustness of the proposed algorithm in [5], ten additional video sequences of people walking in various complicated scenarios in front of different nonuniform backgrounds were collected. Similar to the KTH dataset, most human activities in Weizmann were performed by a single person without any human-object interaction; thus, it is categorized in the activity/action level.

#### 8.2.3. MSR Action3D Dataset (2010)

The MSR Action3D dataset [76] is widely used as the benchmark for depth-based HAR approaches. Depth maps of 20 activity classes performed by 10 subjects are provided in it (high arm waving, horizontal arm waving, hammering, hand catching, forward punching, high throwing, drawing cross, drawing tick, drawing circle, clapping hand, waving two hand, side-boxing, bending, forward kicking, side kicking, jogging, tennis swing, tennis serve, golf swing, pickup, and throw). MSR Action3D is a pure depth datasets without any color images in it.

### 8.3. Interaction Level Datasets

Interaction level datasets are relatively difficult tasks. Due to the human or human-object interactions, interaction level human activities are more realistic and abound in various scenarios such as sport events [91], video surveillance, and different movie scenes [50]. In this section, we review two conventional RGB datasets (i.e., Hollywood human activity dataset and UCF sports human activity dataset) and a RGB-D dataset (i.e., MSR DailyActivity3D dataset). Designed to cover indoor daily activities, MSR DailyActivity3D dataset [160] is more challenging and involves more human-object interactions compared to MSR Action3D [82].

#### 8.3.1. Hollywood Human Activity Dataset (2008 and 2009)

Another well-known interaction level dataset is the Hollywood human activity dataset [50, 158]. As a representative of realistic activity dataset, the Hollywood dataset is introduced here as a challenging task compared to previous datasets due to its frequently moved camera viewpoints, occlusions, and dynamic backgrounds with seldom provided information [1]. The initial version published in 2008 [50] contains approximately 663 video samples (233 samples in automatic training set, 219 samples in clean training set, and 211 samples in test set) of eight actions (answering phone, getting out of car, hugging, handshaking, kissing, sitting down, sitting up, and standing up) from 32 movies. Recognition of natural human activities in diverse and realistic video settings, which can be tested on this dataset, was discussed in [50]. Then, the extended Hollywood dataset was created in 2009 [158], involving four additional activities (driving a car, eating, fighting, and running) and more samples for each class, totally, 3669 video clips from 69 movies. Both human interaction (e.g., kissing, fighting) and human-object interactions (e.g., answering phone, driving a car) are included. Marszalek et al. [158] exploited the relationship between context of natural dynamic scenes and human activities in video based on this extended Hollywood dataset.

#### 8.3.2. UCF Sports Dataset (2007)

The UCF sports dataset [91] is a specific interaction level dataset focused on various sports activities from television broadcasts. It is one of the datasets collected by Computer Vision Lab, University of Central Florida. There are over 200 video sequences in this dataset, covering 9 sport activities including diving, golf swinging, kicking, lifting, horseback riding, running, skating, swinging a basketball bat, and pole vaulting. While it covers only 9 human activities in sports scenes, it is still a challenging task for recognition due to its unconstrained environment and abound intraclass variability.

#### 8.3.3. MSR DailyAction3D Dataset (2012)

The MSR DailyActivity3D dataset [160] is an interactive level dataset captured by Kinect device. In contrast with the previous MSR Action3D, this dataset provides three types of data including depth maps, skeleton joint positions, and RGB video. 16 activity classes performed by 10 subjects (drinking, eating, reading book, calling cellphone, writing on a paper, using laptop, using vacuum cleaner, cheering up, sitting still, tossing paper, playing game, lying down on sofa, walking, playing guitar, standing up, and sitting down) are recorded in it.

## 9. Conclusions and Future Direction

Human activity recognition remains to be an important problem in computer vision. HAR is the basis for many applications such as video surveillance, health care, and human-computer interaction. Methodologies and technologies have made tremendous development in the past decades and have kept developing up to date. However, challenges still exist when facing realistic sceneries, in addition to the inherent intraclass variation and interclass similarity problem.

In this review, we divided human activities into three levels including action primitives, actions/activities, and interactions. We have summarized the classic and representative approaches to activity representation and classification, as well as some benchmark datasets in different levels. For representation approaches, we roughly sorted out the research trajectory from global representations to local representations and recent depth-based representations. The literatures were reviewed in this order. State-of-the-art approaches, especially those depth-based representations, were discussed, aiming to cover the recent development in HAR domain. As the next step, classification methods play important roles and prompt the advance of HAR. We categorized classification approaches into template-matching methods, discriminative models, and generative models. Totally, 7 types of method from the classic DTW to the newest deep learning were summarized. For human tracking approaches, two categories are considered namely filter-based and kernel-based human tracking. Finally, 7 datasets were introduced, covering different levels from primitive level to interaction level, ranging from classic datasets to recent benchmark for depth-based methods.

Though recent HAR approaches have achieved great success up to now, applying current HAR approaches in real-world systems or applications is still nontrivial. Three future directions are recommended to be considered and further explored.

First, current well-performed approaches are mostly hard to be implemented in real time or applied to wearable devices, as they are subject to constrained computing power. It is difficult for computational constrained systems to achieve comparable performances of those offline approaches. Existing work utilized additional inertial sensors to assist in recognizing, or developed microchips, for embedded devices. Besides these hardware-oriented solutions, from a computer vision perspective, more efficient descriptor extracting methods and classification approaches are expected to train recognition models fast, even in real time. Another possible way is to degrade quality of input image and strike a balance among input information, algorithm efficiency, and recognizing rate. For example, utilizing depth maps as inputs and abandoning color information are ways of degrading quality.

Second, many of the recognition tasks are solved case by case, for both the benchmark datasets and the recognition methods. The future direction of research is obviously encouraged to unite various datasets as a large, complex, and complete one. Though every dataset may act as benchmark in its specific domain, uniting all of them triggers more effective and general algorithms which are more close to real-world occasions. For example, recent deep learning is reported to perform better in a four-dataset-combined larger datasets [114]. Another promising direction is to explore an evaluation criterion which enables comparisons among wide variety of recognition methods. Specifically, several vital measuring indexes are defined and weighted according to specific task, evaluating methods by measuring indexes such as recognition rate, efficiency, robustness, number, and level of recognizable activities.

Third, mainstream recognition system remains in a relatively low level comparing with those higher level behaviors. Ideally, the system should be able to tell the behavior “having a meeting” rather than lots of people sitting and talking, or even more difficult, concluding that a person hurried to catch a bus rather than just recognizing “running.” Activities are analogous to the words consisting behavior languages. Analyzing logical and semantic relations between behaviors and activities is an important aspect, which can be learned by transferring from Natural language processing (NLP) techniques. Another conceivable direction is to derive additional features from contextual information. Though this direction has been largely exploited, current approaches usually introduce all the possible contextual variables without screening. This practice not only reduces the efficiency but also affects the accuracy. Thus, dynamically and reasonably choosing contextual information is a future good topic to be discussed.

Finally, though recent deep learning approaches achieve remarkable performance, a conjoint ConvNets + LSTM architecture is expected for activity video analysis in the future. On the one hand, ConvNets are spatial extension of conventional neural networks and exhibit its advantage in the image classification tasks. This structure captures the spatial correlation characteristics, however, ignores the temporal dependencies of the interframe content for activity dynamics modeling. On the other hand, LSTM as a representative kind of RNN, is able to model the temporal or sequence information, which makes up the temporal shortage of ConvNets. LSTM is currently used in accelerometer-based recognition, skeleton-based activity recognition, or one-dimensional signal processing, but has not been widely concerned in combination with ConvNets for two-dimensional video activity recognition, which we believe is a promising direction in the future.

## Figures and Tables

**Table 1:** Taxonomy of activity recognition literatures.

References	Year	Representation (global/local/depth)	Classification	Modality	Level	Dataset	Performance result
Yamato et al. [94]	1992	Symbols converted from mesh feature vector and encoded by vector quantization (G)	HMM	RGB	Action/activity	Collected dataset: 3 subjects × 300 combinations	96% accuracy
Darrell and Pentland [92]	1993	View model sets (G)	Dynamic time warping	RGB	Action primitive	Collected instances of 4 gestures.	96% accuracy (“Hello” gesture)
Brand et al. [102]	1997	2D blob feature (G)	Coupled HMM (CHMM)	RGB	Action primitive	Collected dataset: 52 instances. 3 gestures × 17 times.	94.2% accuracy
Oliver et al. [97]	2000	2D blob feature (G)	(i) CHMM; (ii) HMM;	RGB	Interaction	Collected dataset: 11–75 training sequences +20 testing sequences. Organized as 5-level hierarchical interactions.	(i) 84.68 accuracy (average); (ii) 98.43 accuracy (average)
Bobick and Davis [17]	2001	Motion energy image & motion history image (G)	Template matching by measuring Mahalanobis distance	RGB	Action/activity	Collected dataset: 18 aerobic exercises × 7 views.	(a) 12/18 (single view); (b) 15/18 (multiple views)
Efros et al. [10]	2003	Optical flow (G)	K-nearest neighbor	RGB	Action/activity	(a) Ballet dataset; (b) tennis dataset; (c) football dataset	(a) 87.4% accuracy; (b) 64.3% accuracy; (c) 65.4% accuracy
Park and Aggarwal [103]	2004	Body model by combining an ellipse representation and a convex hull-based polygonal representation (G)	Dynamic Bayesian network	RGB	Interaction	Collected dataset: 56 instances. 9 interactions × 6 pairs of people.	78% accuracy
Schüldt et al. [105]	2004	Space-time interest points (L)	SVM	RGB	Action/activity	KTH dataset	71.7% accuracy
Blank et al. [5]	2005	Space-time shape (G)	Spectral clustering algorithm	RGB	Action/activity	Weizmann dataset	99.63% accuracy
Oikonomopoulos et al. [36]	2005	Spatiotemporal salient points (L)	RVM	RGB	Action/activity	Collected dataset: 152 instances. 19 activities × 4 subjects × 2 times.	77.63% recall
Dollar et al. [37]	2005	Space-time interest points (L)	(i) 1-nearest neighbor (1NN); (ii) SVM;	RGB	Action/activity	KTH dataset	(i) 78.5% accuracy (1NN); (ii) 81.17% accuracy (SVM)
Ke et al. [38]	2005	Integral videos (L)	Adaboost	RGB	Action/activity	KTH dataset	62.97% accuracy
Veeraraghavan et al. [93]	2005	Space-time shape (G)	Nonparametric methods by extending DTW	RGB	Action/activity	(a) USF dataset [154]; (b) CMU dataset [155]; (c) MOCAP dataset	No accuracy data presented.
Duong et al. [98]	2005	High level activities are represented as sequences of atomic activities; atomic activities are only represented using durations (−).	Switching hidden semi-Markov model (S-HSMM)	RGB	Interaction	Collected dataset: 80 video sequences. 6 high level activities.	97.5 accuracy (average accuracy; Coxian model)
Weinland et al. [20]	2006	Motion history volumes (G)	Principal component analysis (PCA) + Mahalanobis distance	RGB	Action/activity	IXMAS dataset [20]	93.33% accuracy
Lu et al. [49]	2006	PCA-HOG (L)	HMM	RGB	Action/activity	(a) Soccer sequences dataset [10]; (b) Hockey sequences dataset [156]	The implemented system can track subjects in videos and recognize their activities robustly. No accuracy data presented.
Ikizler and Duygulu [18]	2007	Histogram of oriented rectangles and encoded with BoVW (G)	(i) Frame by frame voting; (ii) global histogramming; (iii) SVM classification; (iv) dynamic time warping;	RGB	Action/activity	Weizmann dataset	100% accuracy (DTW)
Huang and Xu [19]	2007	Envelop shape acquired from silhouettes (G)	HMM	RGB	Action/activity; action primitive	Collected dataset: 9 activities × 7 subjects × 3 times × 3 views.	Subject dependent + view independent: 97.3% accuracy; subject independent + view independent: 95.0% accuracy; subject independent + view dependent: 94.4% accuracy
Scovanner et al. [46]	2007	3D SIFT (L)	SVM	RGB	Action/activity	Weizmann dataset	82.6% accuracy
Vail et al. [106]	2007	—	(i) HMM (ii) conditional random field	—	Interaction	Data from the hourglass and the unconstrained tag domains generated by robot simulator.	98.1% accuracy (CRF, hourglass); 98.5% accuracy (CRF, unconstrained tag domains)
Cherla et al. [21]	2008	Width feature of normalized silhouette box (G)	Dynamic time warping	RGB	Action/activity	IXMAS dataset [20]	80.05% accuracy; 76.28% accuracy (cross view)
Tran and Sorokin [25]	2008	Silhouette and optical flow (G)	(i) Naïve Bayes (NB); (ii) 1-nearest neighbor (1NN); (iii) 1-nearest neighbor with rejection (1NN-R); (iv) 1-nearest neighbor with metric learning (1NN-M)	RGB	Interaction; Action/activity	(a) Weizmann dataset; (b) UMD dataset [15]; (c) IXMAS dataset [20]; (d) collected dataset: 532 instances. 10 activities × 8 subjects.	(a) 100% accuracy; (b) 100% accuracy; (c) 81% accuracy; (d) 99.06% accuracy (1NN-M & L1SO)
Achard et al. [26]	2008	Semi-global features extracted from space-time micro volumes (L)	HMM	RGB	Action/activity	Collected dataset: 1614 instances. 8 activities × 7 subjects × 5 views.	87.39% accuracy (average)
Rodriguez et al. [91]	2008	Action MACH-maximum average correlation height (G)	Maximum average correlation height filter	RGB	Interaction; Action/activity	(a) KTH dataset; (b) collected feature films dataset: 92 kissing + 112 hitting/Slapping; (c) UCF dataset; (d) Weizmann dataset	(a) 80.9% accuracy; (b) 66.4% for kissing & 67.2% for hitting/slapping; (c) 69.2% accuracy; (d) reported a significant increase in algorithm efficiency, with no overall accuracy data presented
Kiaser et al. [30]	2008	Histograms of oriented 3D spatiotemporal gradients (L)	SVM	RGB	Interaction; Action/activity	(a) KTH dataset; (b) Weizmann dataset; (c) Hollywood dataset	(a) 91.4% (±0.4) accuracy; (b) 84.3% (±2.9) accuracy; (c) 24.7% precision
Willems et al. [39]	2008	Hessian-based STIP detector & SURF3D (L)	SVM	RGB	Action/activity	KTH dataset	84.26% accuracy
Laptev et al. [50]	2008	STIP with HOG, HOF are encoded with BoVW (L)	SVM	RGB	Interaction; Action/activity	(a) KTH dataset; (b) Hollywood dataset	(a) 91.8% accuracy; (b) 38.39% accuracy (average)
Natarajan and Nevatia [95]	2008	23 degrees body model (G)	Hierarchical variable transition HMM (HVT-HMM)	RGB	Action/activity; Action primitive	(a) Weizmann dataset; (b) gesture dataset in [157]	(a) 100% accuracy; (b) 90.6% accuracy
Natarajan and Nevatia [107]	2008	2-layer graphical model: top layer corresponds to actions in particular viewpoint; lower layer corresponds to individual poses (G)	Shape, flow, duration-conditionalrandom field (SFD-CRF)	RGB	Action/activity	Collected dataset: 400 instances. 6 activities × 4 subjects × 16 views (×6 backgrounds).	78.9% accuracy
Ning et al. [108]	2008	Appearance and position context (APC) descriptor encoded by BoVW (L)	Latent pose conditional random fields (LPCRF)	RGB	Action/activity; Action primitive	HumanEva dataset	95.0% accuracy (LPCRF*init*)
Marszalek et al. [158]	2009	SIFT, HOG, HOF encoded by BoVW (L)	SVM	RGB	Interaction	Hollywood2 dataset	35.5% accuracy
Li et al. [76]	2010	Action graph of salient postures (D)	Non-Euclidean relational fuzzy (NERF) C-means & Hausdorf distance-based dissimilarity measure	Depth	Action/activity	MSR Action3D dataset	91.6% accuracy (train/test = 1/2); 94.2% accuracy (train/test = 2/1); 74.7% accuracy (train/test = 1/1 & cross subject)
Suk et al. [101]	2010	YIQ color model for skin pixels; histogram-based color model for face region; optical flow for tracking of hand motion (L)	Dynamic Bayesian network	RGB	Action primitive	Collected dataset: 498 instances. (a) 10 gestures × 7 subjects × 7 times (isolated gesture); (b) 8 longer videos contain 50 gestures (continuous gestures)	(a) 99.59% accuracy; (b) 84% recall & 80.77% precision
Baccouche et al. [124]	2010	SIFT descriptor encoded by BoVW (L)	Recurrent neural networks (RNN) with long short-term memory (LSTM)	RGB	Interaction	MICC-Soccer-Actions-4 dataset [159]	92% accuracy
Kumari and Mitra [29]	2011	Discrete Fourier transform on silhouettes (G)	K-nearest neighbor	RGB	Action/activity	(a) MuHaVi dataset; (b) DA-IICT dataset;	(a) 96% accuracy; (b) 82.6667% accuracy;
Wang et al. [51]	2011	Dense trajectory with HOG, HOF, MBH (L)	SVM	RGB	Interaction; Action/activity	(a) KTH dataset; (b) YouTube dataset; (c) Hollywood2 dataset; (d) UCF Sport dataset	(a) 94.2% accuracy; (b) 84.2% accuracy; (c) 58.3% accuracy; (d) 88.2% accuracy
Wang et al. [56]	2012	STIP with HOG, HOF are encoded with various encoding methods (L)	SVM	RGB	Interaction; Action/activity	(a) KTH dataset; (b) HMDB51 dataset	(a) 92.13% accuracy (Fisher vector); (b) 29.22% accuracy (Fisher vector)
Zhao et al. [77]	2012	Combined representations: (a) RGB: HOG & HOF upon space-time interest points (L) (b) depth: local depth pattern at each interest point (D)	SVM	RGB-D	Interaction	RGBD-HuDaAct dataset	89.1% accuracy
Yang et al. [78]	2012	DMM-HOG (D)	SVM	Depth	Action/activity	MSR Action3D dataset	95.83% accuracy (train/test = 1/2); 97.37% accuracy (train/test = 2/1); 91.63% accuracy (train/test = 1/1 & cross subject)
Xia et al. [84]	2012	Histograms of 3D joint locations (D)	HMM	Depth	Action/activity	(a) collected dataset: 6220 frames, 200 samples. 10 activities × 10 subjects × 2 times. (b) MSR Action3D dataset	(a) 90.92% accuracy; (b) 97.15% accuracy (highest); 78.97% accuracy (cross subject)
Yang and Tian [85]	2012	EigenJoints (D)	Naïve-Bayes-Nearest-Neighbor (NBNN)	Depth	Action/activity	MSR Action3D dataset	96.8% accuracy; 81.4% accuracy (cross subject)
Wang et al. [160]	2012	Local occupancy pattern for depth maps & Fourier temporal pyramid for temporal representation & actionlet ensemble model for characterizing activities (D)	SVM	Depth	Interaction; Action/activity	(a) MSR Action3D dataset; (b) MSR Action3DExt dataset; (c) CMU MOCAP dataset	(a) 88.2% accuracy; (b) 85.75% accuracy; (c) 98.13% accuracy
Wang et al. [53]	2013	Improved dense trajectory with HOG, HOF, MBH (L)	SVM	RGB	Interaction	(a) Hollywood2 dataset; (b) HMDB51 dataset; (c) Olympic Sports dataset [161]; (d) UCF50 dataset [162]	(a) 64.3% accuracy; (b) 57.2% accuracy; (c) 91.1% accuracy; (d) 91.2% accuracy
Oreifej and Liu [74]	2013	Histogram of oriented 4D surface normals (D)	SVM	Depth	Action/activity; Action primitive	(a) MSR Action3D dataset; (b) MSR Gesture3D dataset; (c) Collected 3D Action Pairs dataset	(a) 88.89% accuracy; (b) 92.45% accuracy; (c) 96.67% accuracy
Chaaraoui [88]	2013	Combined representations: (a) RGB: silhouette (G) (b) depth: skeleton joints (D)	Dynamic time warping	RGB-D	Action/activity	MSR Action3D dataset	91.80% accuracy
Ren et al. [152]	2013	Time-series curve of hand shape (G)	Dissimilarity measure based on Finger-Earth Mover's Distance (FEMD)	RGB	Action primitive	Collected dataset: 1000 instances. 10 gestures × 10 subjects × 10 times.	93.9% accuracy
Ni et al. [163]	2013	Depth-Layered Multi-Channel STIPs (L)	SVM	RGB-D	Interaction	RGBD-HuDaAct database	81.48% accuracy (codebook size = 512 & SPM kernel)
Grushin et al. [123]	2013	STIP with HOF (L)	Recurrent neural networks (RNN) with long short-term memory (LSTM)	RGB	Action/activity	KTH dataset	90.7% accuracy
Peng et al. [31]	2014	(i) STIP with HOG, HOF and encoded by various encoding methods; (L) (ii) iDT with HOG, HOF, MBHx, MBHy and encoded by various encoding methods (L)	SVM	RGB	Interaction	(a) HMDB51 dataset; (b) UCF50 dataset; (c) UCF101 dataset	Hybrid representation: (a) 61.1% accuracy; (b) 92.3% accuracy; (c) 87.9% accuracy
Peng et al. [32]	2014	Improved dense trajectory encoded with stacked Fisher kernal (L)	SVM	RGB	Interaction; Action/activity	(a) YouTube dataset; (b) HMDB51 dataset; (c) J-HMDB dataset	(a) 93.38% accuracy; (b) 66.79% accuracy; (c) 67.77% accuracy
Wang et al. [82]	2014	Local occupancy pattern for depth maps & Fourier temporal pyramid for temporal representation & actionlet ensemble model for characterizing activities (D)	SVM	Depth	Interaction; Action/activity	(a) MSR Action3D dataset; (b) MSR DailyActivity3D dataset; (c) Multiview 3D event dataset; (d) Cornell Activity Dataset [164]	(a) 88.2% accuracy; (b) 85.75% accuracy; (c) 88.34% accuracy (cross subject); 86.76% accuracy (cross view); (d) 97.06% (same person) 74.70% accuracy (cross person)
Simonyan and Zisserman [115]	2014	Spatial stream ConvNets & optical flow based temporal stream ConvNets (L)	SVM	RGB	Interaction	(a) HMDB51 dataset; (b) UCF101 dataset	(a) 59.4% accuracy; (b) 88.0% accuracy
Lan et al. [33]	2015	Improved dense trajectory with HOG, HOF, MBHx, MBHy enhanced with multiskip feature tracking (L)	SVM	RGB	Interaction	(a) HMDB51 dataset; (b) Hollywood2 dataset; (c) UCF101 dataset; (d) UCF50 dataset; (e) Olympic Sports dataset	(a) 65.1% accuracy (L = 3); (b) 68.0% accuracy (L = 3); (c) 89.1% accuracy (L = 3); (d) 94.4% accuracy (L = 3); (e) 91.4% accuracy (L = 3)
Shahroudy et al. [83]	2015	Combined representations: (a) RGB: dense trajectories with HOG, HOF, MBH (L) (b) Depth: skeleton joints (D)	SVM	RGB-D	Interaction	MSR DailyActivity3D	81.9% accuracy
Wang et al. [114]	2015	Weighted hierarchical depth motion maps (D)	Three-channel deep convolutional neural networks (3ConvNets)	Depth	Interaction; Action/activity	(a) MSR Action3D dataset; (b) MSR Action3DExt dataset; (c) UTKinect Action dataset [84]; (d) MSR DailyActivity3D dataset; (e) Combined dataset of above	(a) 100% accuracy; (b) 100% accuracy; (c) 90.91% accuracy; (d) 85% accuracy; (e) 91.56% accuracy
Wang et al. [165]	2015	Pseudo-color images converted from DMMs (D)	Three-channel deep convolutional neural networks (3ConvNets)	Depth	Interaction; Action/activity	(a) MSR Action3D dataset; (b) MSR Action3DExt dataset; (c) UTKinect Action dataset [84]	(a) 100% accuracy; (b) 100% accuracy; (c) 90.91% accuracy
Wang et al. [117]	2015	Trajectory-pooled deep-convolutional descriptor and encoded by Fisher kernal (L)	SVM	RGB	Interaction	(a) HMDB51 dataset; (b) UCF101 dataset	(a) 65.9% accuracy; (b) 91.5% accuracy
Veeriah et al. [125]	2015	(i) HOG3D in KTH 2D action dataset; (L) (ii) skeleton-based features including skeleton positions, normalized pair-wise angels, offset of joint positions, histogram of the velocity, and pairwise joint distances (D)	Differential recurrent neural network (dRNN)	RGBD	Action/activity	(a) KTH dataset; (b) MSR Action3D dataset	(a) 93.96% accuracy (KTH-1); 92.12% accuracy (KTH-2); (b) 92.03% accuracy
Du et al. [126]	2015	Representations of skeleton data extracted by subnets (D)	Hierarchical bidirectional recurrent neural network (HBRNN)	RGBD	Action/activity	(a) MSR Action3D dataset; (b) Berkeley MHAD Action dataset [166]; (c) HDM05 dataset [167]	(a) 94.49% accuracy; (b) 100% accuracy; (c) 96.92% (±0.50) accuracy
Zhen et al. [58]	2016	STIP with HOG3D and encoded with various encoding methods (L)	SVM	RGB	Interaction; Action/activity	(a) KTH dataset; (b) UCF YouTube dataset; (c) HMDB51 dataset	(a) 94.1% (Local NBNN); (b) 63.0% (improved Fisher kernal); (c) 30.5% (improved Fisher kernal)
Chen et al. [81]	2016	Action graph of skeleton-based features (D)	Maximum likelihood estimation	Depth	Action/activity	(a) MSR Action3D dataset; (b) UTKinect Action dataset	(a) 95.56% accuracy (cross subject); 96.1% accuracy (three subset evaluation); (b) 95.96% accuracy
Zhu et al. [87]	2016	Co-occurrence features of skeleton joints (D)	Recurrent neural networks (RNN) with long short-term memory (LSTM)	Depth	Interaction; Action/activity	(a) SBU Kinect interaction dataset [168]; (b) HDM05 dataset; (c) CMU dataset; (d) Berkeley MHAD Action dataset	(a) 90.41% accuracy; (b) 97.25% accuracy; (c) 81.04% accuracy; (d) 100% accuracy
Li et al. [116]	2016	VLAD for deep dynamics (G)	Deep convolutional neural networks (ConvNets)	RGB	Interaction; Action/activity	(a) UCF101 dataset; (b) Olympic Sports dataset; (c) THUMOS15 dataset [116]	(a) 84.65% accuracy; (b) 90.81% accuracy; (c) 78.15% accuracy
Berlin & John [119]	2016	Harris corner-based interest points and histogram-based features (L)	Deep neural networks (DNNs)	RGB	Interaction	UT Interaction dataset [169]	95% accuracy on set1; 88% accuracy on set2
Huang et al. [120]	2016	Lie group features (L)	Lie Group Network (LieNet)	Depth	Interaction; Action/activity	(a) G3D-Gamingdataset [170]; (b) HDM05 dataset; (c) NTU RGBD dataset [171]	(a) 89.10% accuracy; (b) 75.78% ± 2.26 accuracy; (c) 66.95% accuracy
Mo et al. [113]	2016	Automatically extracted features from skeletons data (D)	Convolutional neural networks (ConvNets) + multilayer perceptron	Depth	Interaction	CAD-60 dataset	81.8% accuracy
Shi et al. [55]	2016	Three stream sequential deep trajectory descriptor (L)	Recurrent neural networks (RNN) and deep convolutional neural networks (ConvNets)	RGB	Interaction; Action/activity	(a) KTH dataset; (b) HMDB51 dataset; (c) UCF 101 dataset [172]	(a) 96.8% accuracy; (b) 65.2% accuracy; (c) 92.2% accuracy
Yang et al. [79]	2017	Low-level polynormal assembled from local neighboring hypersurface normals and are then aggregated by Super Normal Vector (D)	Linear classifier	Depth	Interaction; Action/activity; Action primitive	(a) MSR Action3D dataset; (b) MSR Gesture3D dataset; (c) MSR ActionPairs3D dataset [173]; (d) MSR DailyActivity3D dataset	(a) 93.45% accuracy; (b) 94.74% accuracy; (c) 100% accuracy; (d) 86.25% accuracy
Jalal et al. [80]	2017	Multifeatures extracted from human body silhouettes and joints information (D)	HMM	Depth	Interaction; Action/activity	(a) Online self-annotated dataset [174]; (b) MSR DailyActivity3D dataset; (c) MSR Action3D dataset	(a) 71.6% accuracy; (a) 92.2% accuracy; (a) 93.1% accuracy

**Table 2:** Feature encoding methods.

Method	Proposed	Description paper, the number of citations
Vector quantization (VQ)/hard assignment (HA)	Sivic et al. (2003)	[59], 5487
Kernal codebook coding (KCB)/soft assignment (SA)	Gemert et al. (2008)	[60], 586; [61], 761
Spase coding (SPC)	Yang et al. (2009)	[62], 2529
Local coordinate coding (LCC)	Yu et al. (2009)	[63], 614
Locality-constrained linear coding (LLC)	Wang et al. (2010)	[64], 2410
Improved Fisher kernel (iFK)/Fisher vector (FV)	Perronnin et al. (2010)	[65], 1590
Triangle assignment coding (TAC)	Coates et al. (2010)	[66], 976
Vector of locally aggregated descriptors (VLAD)	Jegou et al. (2010)	[67], 1135; [68], 710;
Super vector coding (SVC)	Zhou et al. (2010)	[69], 459
Local tangent-based coding (LTC)	Yu et al. (2010)	[70], 122
Localized soft assignment coding (LSC/SA-*k*)	Liu et al. (2011)	[71], 398
Salient coding (SC)	Huang et al. (2011)	[72], 131
Group salient coding (GSC)	Wu et al. (2012)	[73], 33
Stacked Fisher vectors (SFV)	Peng et al. (2014)	[32], 149

**Table 3:** Overview of representative datasets.

Dataset	Modality	Level	Year	References	Web pages	Activity category
RGBD-HuDaAct	RGB-D	Interaction level	2013	[163]	http://adsc.illinois.edu/sites/default/files/files/ADSC-RGBD-dataset-download-instructions.pdf	12 classes: eat meal, drink water, mop floor, and so forth
Hollywood	RGB	Interaction level	2008	[50]	http://www.di.ens.fr/~laptev/download.html#actionclassification	8 classes: answer phone, hug person, kiss, and so forth
Hollywood-2	RGB	Interaction level	2009	[158]	http://www.di.ens.fr/~laptev/download.html#actionclassification	12 classes: answer phone, driving a car, fight, and so forth
UCF sports	RGB	Interaction level	2008	[91]	http://crcv.ucf.edu/data/UCF_Sports_Action.php	10 classes: golf swing, diving, lifting, and so forth
KTH	RGB	Activity/action level	2004	[105]	http://www.nada.kth.se/cvap/actions/	6 classes: walking, jogging, running, and so forth
Weizmann	RGB	Activity/action level	2005	[5]	http://www.wisdom.weizmann.ac.il/~vision/SpaceTimeActions.html	10 classes: run, walk, bend, jumping-jack, and so forth
NTU-MSR	RGB-D	Action primitive level	2013	[152]	http://web.cs.ucla.edu/~zhou.ren/	10 classes: it contains 10 different gestures.
MSRC-Gesture	RGB-D	Action primitive level	2012	[153]	http://research.microsoft.com/en-us/um/cambridge/projects/msrc12/	12 classes: it contains 12 different gestures.
MSR DailyAction3D	RGB-D	Interaction level	2012	[160]	http://research.microsoft.com/en-us/um/people/zliu/actionrecorsrc/default.htm	16 classes: call cellphone, use laptop, walk, and so forth
MSR Action3D	Depth	Activity/action level	2010	[76]	http://research.microsoft.com/en-us/um/people/zliu/actionrecorsrc/default.htm	20 classes: high arm wave, hand clap, jogging, and so forth
